# Whole-Genome Sequence of the Cyanobacterium *Synechococcus* sp. Strain WH 8101

**DOI:** 10.1128/MRA.01593-19

**Published:** 2020-02-20

**Authors:** Marcia F. Marston, Shawn W. Polson

**Affiliations:** a Department of Biology and Marine Biology, Roger Williams University, Bristol, Rhode Island, USA; b Center for Bioinformatics and Computational Biology, Delaware Biotechnology Institute, University of Delaware, Newark, Delaware, USA; Georgia Institute of Technology

## Abstract

*Synechococcus* spp. are unicellular cyanobacteria that are globally distributed and are important primary producers in marine coastal environments. Here, we report the complete genome sequence of *Synechococcus* sp. strain WH 8101 and identify genomic islands that may play a role in virus-host interactions.

## ANNOUNCEMENT

*Synechococcus* spp. are responsible for up to 16% of net primary production in the oceans (1). Significant proportions of marine *Synechococcus* communities can be lysed daily by viruses (2, 3); nevertheless, studies suggest that *Synechococcus* strains can rapidly become resistant to co-occurring viruses (4, 5). In an effort to identify the genetic determinants that lead to viral resistance, the complete genome of *Synechococcus* sp. strain WH 8101 was sequenced.

*Synechococcus* sp. strain WH 8101 was obtained from F. W. Valois, who isolated it in 1981 from surface seawater collected at Woods Hole, Massachusetts (41°31ʹ34ʺN, 70°40ʹ13ʺW), as described previously (6). The strain has been maintained in SN medium since isolation (6). Based on multiple DNA markers and physiological characteristics, WH 8101 has been assigned to *Synechococcus* clade VIII (7, 8). Only one other member of this clade (*Synechococcus* sp. strain RS9917) has been sequenced.

A single colony of WH 8101 was isolated on an SN soft-agar plate and then regrown in SN medium prior to DNA isolation (6). Genomic DNA was sequenced using both Illumina MiSeq and PacBio RS II platforms. For Illumina sequencing, DNA was isolated using the PowerWater DNA isolation kit (MoBio Laboratories), and a DNA library was prepared using the WaferGen Apolla 324 next-generation sequencing library preparation system with an IntegenX PrepX DNA library kit. The library was sequenced on the Illumina MiSeq system using the 500-cycle reagent kit v.2. For PacBio sequencing, DNA was isolated using the Genomic-tip 100/G kit (Qiagen), libraries were prepared using the standard PacBio 20-kb protocol, and fragments were size selected (>10 kb) with BluePippin (Sage Science) and sequenced on a PacBio RS II system in one single-molecule real-time (SMRT) cell, using P6-C4 chemistry (6-h movie). Reads (50,981 reads; *N*_50_, 20,257 bp) were filtered (>750 bp) and assembled using HGAP.3 (seed cutoff, 6 kb). The consensus sequence was polished by additional rounds of PacBio read mapping and was circularized using information from the bridge mapper tool, all within the SMRT Analysis software (v.2.3.0.140936), using default settings. MiSeq reads were mapped to the initial PacBio assembly using Geneious v.10 with default settings and used for additional quality control and manual correction of indel errors. Coverages were 45× and 175× for the MiSeq and PacBio reads, respectively. A single circular 2,630,292-bp assembly with a G+C content of 63.3% was obtained. The genome was initially annotated using RASTtk (9) and subsequently updated with the NCBI Prokaryotic Genome Annotation Pipeline (NCBI RefSeq database). The genome includes 2,693 protein-coding genes, 41 pseudogenes, 6 rRNAs, and 43 tRNAs.

Genes for viral resistance are often localized to genomic islands (hypervariable regions) in *Synechococcus* and *Prochlorococcus* spp. (4, 10). Using previously established criteria (10, 11), 13 genomic islands were identified in WH 8101 (Table 1). These regions were >8 kb and/or contained at least 10 genes that were not in synteny with the genome of the other clade VIII strain, *Synechococcus* sp. strain RS9917. Genomic islands that were identified in RS9917 (11) and present in WH 8101 were also included. This genomic sequence will be used to identify genetic determinants of cyanophage resistance.

**TABLE 1:** Genomic islands in *Synechococcus* sp. WH 8101

Island	Genomic location (nucleotides, start to stop)	Length (nucleotides)	No. of genes	Comparison to strain RS9917^*a*^
ISL1	45853 to 54972	9,120	9	Insertion in WH 8101
ISL2	214977 to 295023	80,047	55	ISL7 in RS9917; 29 common genes
ISL3	624692 to 634958	10,267	9	Insertion in WH 8101
ISL4	655863 to 667839	11,977	14	Same genes as ISL5 in RS9917
ISL5	764807 to 811294	46,488	40	ISL4 in RS9917; 13 common genes
ISL6	959925 to 1050615	90,691	100	ISL2 in RS9917; 6 common genes
ISL7	1142010 to 1223399	81,390	125	ISL13 in RS9711; 6 common genes
ISL8	1256253 to 1278602	22,350	28	ISL12 in RS9917; 20 common genes
ISL9	1289127 to 1322908	33,782	23	Insertion in WH 8101
ISL10	1588101 to 1721246	133,146	153	ISL10 in RS9917; 71 common genes
ISL11	1886863 to 1966465	79,603	65	ISL9 in RS9117; 39 common genes
ISL12	2264944 to 2273061	8,118	7	ISL8 in RS9917; 4 common genes
ISL13	2572188 to 2582000	9,813	11	Insertion in WH 8101

a Islands in *Synechococcus* sp. strain RS9917 were identified by Dufresne et al. (11).

### Data availability.

The complete genome sequence of *Synechococcus* sp. strain WH 8101 has been deposited in GenBank (accession number NZ_CP035914), along with raw sequence and methylation data (accession number PRJNA518918).
